# *Escherichia coli* O157:H7/NM prevalence in raw beef, camel, sheep, goat, and water buffalo meat in Fars and Khuzestan provinces, Iran

**Published:** 2012

**Authors:** Ebrahim Rahimi, Hamid Reza Kazemeini, Mohammad Salajegheh

**Affiliations:** 1 *Department of Food Hygiene, College of Veterinary Medicine, Shahrekord Branch, Islamic Azad University, Shahrekord, Iran; *; 2 *Young Researchers Club, Shahrekord Branch, Islamic Azad University, Shahrekord, Iran;*; 3 *Graduated of Veterinary Medicine, College of Veterinary Medicine, Shahrekord Branch, Islamic Azad University, Shahrekord, Iran. *

**Keywords:** *Escherichia coli* O157:H7/NM, Raw meat, Enterohemorrhagic *Escherichia coli*, Iran

## Abstract

Enterohemorrhagic *Escherichia coli* (EHEC) of the O157:H7 serotype is a worldwide zoonotic pathogen responsible for the majority of severe cases of human EHEC disease. The aim of the present study was to investigate the prevalence of *E. coli* O157: H7/NM in raw meat samples from two provinces of Iran. During a period from March 2010 to March 2011. Two hundred and ninety five raw meat samples were collected from beef (n= 85), camel, (n= 50), sheep (n= 62), goat (n= 60), and water buffalo (n=38). Fourteen (4.7%) of the 295 samples were positive for *E. coli* O157. The highest prevalence of *E. coli* O157 was found in beef samples (8.2%), followed by water buffalo (5.3%), sheep (4.8%), camel (2.0%), and goat (1.7%). Of fourteen *E. coli* O157 isolates, only one was determined to be serotype O157: H7 while 13 were determined as serotype O157: NM. Of the 14 *E. coli* O157:H7/NM isolates, one, four, two, and one strains were positive for *stx1*, *stx2*, *eaeA* and *ehlyA* genes, respectively. The prevalence of this organism varied between seasons with the highest prevalence of *E. coli* O157 occurring in summer (9.3%). The results of this study showed that beef and water buffalo meat are a significant source for human EHEC *E. coli* O157:H7/NM infection in Iran. The data reported in this study provides some useful baseline in formation for future research such as molecular or epidemiologic works.

## Introduction

Enterohemorrhagic *Escherichia coli* (EHEC) causes hemorrhagic colitis which is often associated with devastating or life-threatening systemic manifestations. The most severe sequel, the hemolytic uremic syndrome (HUS), results from shiga toxins (Stxs) produced by the bacteria in the intestine that act systemically on sensitive cells in the kidneys, brain, and other organs.^1^ Although most EHEC strains produce Stxs, those produced by EHEC O157:H7 are particularly virulent and are responsible for the majority of HUS cases of bacterial etiology worldwide.^1^^, ^2 In addition to shiga toxins, the *eaeA* gene that encodes for intimin and the *hly* gene that encodes for hemolysin are other main virulence factors.^3^^,^^4^

Domestic and wild animals are sources of EHEC O157:H7,^5^^,^^6^ but the major animal carriers are healthy domesticated ruminants, primarily cattle^1^ and, to a lesser extent, sheep, and possibly goats.^7^ Contamination of meat with fecal material in the slaughtering process is the main transmission route of *E. coli* O157:H7.^8^ Human infections caused by *E. coli* O157:H7 have been reported in more than 30 countries. Cattle appear to be the chief source of infection; with many outbreaks of the disease being linked to the consumption of beef.^4^ Many studies report that the prevalence rates of *E. coli* O157 or *E. coli* O157:H7 in ground beef and meat from other ruminants range from 0.1 to > 50%. In addition, virulence genes have also been reported to be present in these isolates.^9^^-^^11^

There is limited information regarding the prevalence of *E. coli* O157:H7 in ruminant meat in Iran. This study was conducted to determine the prevalence of *E. coli* O157:H7/NM contamination in retail raw beef, and meat from camel, sheep, goat, and water buffalo in Fars and Khuzestan provinces, Iran.

## Materials and Methods

**Sample collection. **From March 2010 to March 2011, 295 raw non pre-packed meat samples from beef (n = 85), camel (n = 50), sheep (n = 62), goat (n = 60), and water buffalo meat (n= 38) were purchased randomly from selected butcheries in Fars and Khuzestan provinces, Iran. Sections of meat (10cm × 10cm × 3cm) from neck of each carcasses was aseptically removed and placed in separate sterile plastic bags to prevent spilling and cross contamination and were immediately transported to the laboratory in a cooler with ice packs.

**Bacteriological examination.** The microbiological examination commenced within 12 h of sample collection. For each meat sample, 25 g was homogenized with 1 g of the homogenate being added to 5 mL of buffered peptone water (BPW- HiMedia Laboratories, Mumbai, India) and incubated. Cultures were streaked onto MacConkey sorbitol agar (HiMedia Laboratories, Mumbai, India) and the plates incubated overnight at 37 °C. From each plate (one plate for each meat sample), 5 to 10 suspected *E. coli* colonies (sorbitol negative and positive) were selected and sub-cultured onto presumptive diagnostic medium and incubated overnight at 37 °C. All sorbitol negative colonies were tested for the O157 antigen by latex agglutination (Oxoid)^12^ and up to five agglutination positive colonies were taken for PCR analysis.

**DNA extraction.** Purification of DNA was achieved using a genomic DNA purification kit (Fermentas, GmbH, Germany) according to the manufacturer’s instruction and the total DNA was measured at 260 nm optical density according to the method described by Sambrook and Russell.^13^

**Detection of** ***fliCh7*** **gene by** **PCR** **analysis.** All oligonucleotide primers were obtained from a commercial source (Cinna Gen, Iran). In order to determine the H7 (*fliCh7*) gene of *E. coli* O157:H7 strains, PCR analysis was used.^14^ The PCR was performed with primers as described previously^15^ (Table 1) in a final volume of 50 μL containing 1× Reaction Buffer (Fermentas, GmbH, Germany), MgCl_2_ (Fermentas, GmbH, Germany), each of the four deoxynucleoside triphosphates (dNTPs) (Fermentas, GmbH, Germany), Taq DNA polymerase (Fermentas, GmbH, Germany), 0.50 μM of primers and 10 μL DNA. DNA amplification reactions were carried out using a DNA thermal cycler (Master Cycler Gradiant, Eppendrof, Germany) with the following program: one cycle of 2 min at 94 °C, 35 cycles of denaturation at 94 °C for 20 s, annealing at 54 °C for 1 min, and extension at 72 °C for 1 min, with a final extension at 72 °C for 10 min. The PCR products were stained with 1% solution of ethidium bromide and visualized under UV light after gel electrophoresis on 1.5% agarose.

**Detection of virulence factors by multiplex PCR analysis. **The *E. coli* O157:H7/NM isolates were screened for the presence of *stx1* (encoding for Shiga toxin 1), *stx2 *(encoding for Shiga toxin 2),* eaeA* (encoding for intimin),and *ehlyA* (encoding for enterohemolysin) genes using PCR method.^14^^,^^15^ The list of primers and the sizes of the expected PCR products are given in Table 1. According to Fratamico *et al*., multiplex PCR protocol was used to prepare the master mix with a total concentration of 50 μL containing incomplete 1× Reaction Buffer [160 mM (NH4)2SO4, 670 mM Tris-HCl (pH 8.8); 0.1% Tween-20] (Fermentas, GmbH, Germany), 3.0 mM MgCl_2_ (Fermentas, GmbH, Germany), 400 μM each of the four deoxynucleoside triphosphates (dNTPs) (Fermentas, GmbH, Germany), 2.5 U Taq DNA polymerase (Fermentas, GmbH, Germany), 0.50 μM of all primers that were used. Then, 10 μL of DNA was added to reaction mixture. Thermal cycling and gel documentation was carried out as mentioned above. 

**Statistical analysis.** Data were transferred to a Microsoft Excel spreadsheet (Microsoft Corp., Redmond, WA, USA). Using SPSS 16.0 statistical software (SPSS Inc., Chicago, IL, USA), a Pearson chi-square test and Fisher's exact two-tailed test analyses were performed and differences were considered significant at *P* < 0.05.

**Table 1:** Primer sequences and predicted lengths of PCR amplification products.^15^

**Gene**	**Primer**	**Oligonucleotide** **sequence (5-3)**	**Fragment** **size (pb)**
*stx1*	SLT1-F	TGTAACTGGAAAGGTGGAGTATACA	210
	SLT1-R	GCTATTCTGAGTCAACGAAAAATAAC	
*stx2*	SLTII-F	GTTTTTCTTCGGTATCCTATTCC	484
	SLTII-R	GATGCATCTCTGGTCATTGTATTAC	
*ehlyA*	AE22	ATTACCATCCACACAGACGGT	397
	AE20-2	ACAGCGTGGTTGGATCAACCT	
*eaeA*	MFS1-F	ACGATGTGGTTTATTCTGGA	166
	MFS1-R	CTTCACGTCACCATACATAT	
*fli*	FLICH7-F	GCGCTGTCGAGTTCTATCGAGC	625
	FLICH7-R	CAACGGTGACTTTATCGCCATTCC	

## Results

Table 2 shows the prevalence of *E. coli* O157 and *E. coli* O157:H7 isolated from beef, camel, sheep, goat and water buffalo meat in Fars and Khuzestan provinces, Iran. Fourteen (4.7%) of 295 samples were positive for *E. coli* O157. The highest prevalence of *E. coli* O157 was found in beef meat samples (8.2%), followed by water buffalo (5.3%), sheep (4.8%), camel (2.0%), and goat (1.7%). There were not significant differences (*P* > 0.05) in the level of contamination with *E. coli* O157 between beef, camel, sheep, goat, and water buffalo meat samples. No significant differences in the prevalence rates were observed between meat samples isolated in Fars or Khuzestan (*P* > 0.05). Out of 14 *E. coli* O157 isolates, only one was serotype O157: H7 and 13 were serotype O157:NM. 

Out of 14 *E. coli* 157:H7/NM isolates, one, four, two, and one strains were positive for *stx1*, *stx2*, *eaeA,* and *ehlyA* genes, respectively (Table 2).

**Table 2:** Prevalence of *Escherichia coli* O157:H7/NM isolated from raw beef, camel, sheep, goat, and water buffalo meat in Iran

**Samples**	**No. of ** **examined samples **	**No. of ** **positive samples (%)**	**Virulence genes **			
			***Stx***_1_	***Stx***_2_	***eaeA***	***ehlyA***
Beef	85	7 (8.2) *	1	2	1	1
Camel	50	1 (2.0)	0	0	0	0
Sheep	62	3 (4.8)	0	1	0	0
Goat	60	1 (1.7)	0	0	0	0
Buffalo	38	2 (5.3)	0	1	1	0
Total	295	14 (4.7)	1	4	2	1

* Out of 7 *E. coli* O157 isolates, 1 was serotype O157: H7

Table 3 shows the seasonal prevalence of *E. coli* O157 in raw beef, camel, sheep, goat, and water buffalo meat samples in Fars and Khuzestan provinces, Iran. The highest prevalence of *E. coli* O157 occurred in summer (9.3%) followed by fall (6.6%). The prevalence rates of *E. coli* O157 in spring and winter were 1.4%. 

**Table 3:** Seasonal prevalence of *Escherichia coli* O157:H7/NM in raw meat of beef, camel, sheep, goat, and water buffalo in Iran.

**Season**	**Meat samples** *					**Total**
	**Beef**	**Camel**	**Sheep**	**Goat**	**Water buffalo**	
Summer	3/22 (13.6)	0/13 (0.0)	2/15 (13.3)	1/15 (6.7)	1/10 (10.0)	7/75 (9.3)
Fall	3/23 (13.0)	1/11 (9.1)	1/17 (5.7)	0/15 (0.0)	0/10 (0.0)	5/76 (6.6)
Winter	0/20 (0.0)	0/13 (0.0)	0/15 (0.0)	0/15 (0.0)	1/10 (10.0)	1/73 (1.4)
Spring	1/20 (5.0)	0/13 (0.0)	0/15 (0.0)	0/15 (0.0)	0/10 (0.0)	1/71 (1.4)

* Results expressed as the number of *Escherichia coli* O157:H7/NM -positive samples / number of samples analyzed (%).

## Discussion

Due to relative increase in the consumption of camel meat in Iran, it was decided to determine the prevalence of *E. coli* O157: H7 in the camel meat. The results of this study showed that only 2.0% of camel meat samples were positive for *E. coli* O157 and *E. coli* O157:H7 was not isolated from any of the camel meat samples. Similar studies in Iran detected E*. coli* O157 in 1.1% of 94 camel carcass samples.^16^ The results of these studies have shown that camel meat is not an important source for *E. coli* O157 infection, however, monitoring and inspection programmes remain critical for preventing outbreaks of food-borne diseases. The occurrence of *E. coli* O157 in camel has rarely been reported. In a study on camel fecal samples from the United Arab Emirates, *E. coli* O157:H7 was not identified.^17^ Studies in five east African countries on fecal and serum samples from 400 camels failed to detect STEC or anti-*Stx* antibodies.^18^

In the present study, 8.2% and 5.3% of retail beef and water buffalo meat samples respectively were *E. coli* O157-positive. According to the results 1.2% of retail beef meat samples were *E. coli* O157:H7-positive. Out of nine *E. coli* O157:H7/NM isolates from beef and water buffalo meat samples, three strains were positive for *stx1*, *stx2*, *eaeA* and *ehlyA* genes. These findings are comparable with those reported from other studies;^11^^,^^19^^-^^24^ however, they are lower than the prevalence rates reported from the Netherlands (10.4%),^25^ England (13.4%),^10^ USA (28.0%),^26^ and Nigeria (28.0%).^27^

In this study, three (4.8%) and one (1.7%) meat samples from sheep and goats respectively were positive for *E. coli* O157. A single isolate from sheep meat samples was positive for the *stx2* gene. In a recent study in Shiraz (Iran), six *E. coli* O157:H7 isolates were recovered form 159 sheep meat samples representing a prevalence rate of 3.8%.^28^ A study in Egypt found the prevalence of *E. coli* O157 to be 2.5% and 2.0% in sheep and goat meat samples, respectively.^29^ The prevalence of *E. coli* O157:H7/NM in retail sheep and goat meat has been reported to be 0.7-7.3% in the Italy,^30^^,^^31^ 4.0% in Egypt,^32^ 1.5-2.2% in USA,^3^^,^^10^ 0.5% in Australia,^33^ and 1.3% in Australia.^34^

Direct comparison of results of this study with other studies is difficult due to differences in the study methodologies, such as the type of slaughtering, improved enrichment and isolation procedures, differences in sample size, the type of sample and how and when it was collected.^35^ While there is some evidence that *E. coli* O157:H7 may be increasingly common in beef production systems the detection of higher proportions of *E. coli* O157:H7 in more recent studies is more probably associated with the wider use of more sensitive detection methods such as Immunomagnetic separation (IMS).^10^

In this study the highest prevalence of *E. coli* O157:H7/NM was found on meat sampled in summer and fall, which is in agreement with findings from previous studies on beef and sheep that reported peak prevalence occurs in summer and early fall.^10^^,^^16^^,^^26^^,^^35^ However, in the only study of non-O157 STEC performed on sheep,^36^ the prevalence did not follow the seasonal trend previously reported for STEC O157:H7, with the highest prevalence rates (up to 26.0%) during winter and spring. Kudva *et al*. hypothesized that changes in diet and/or environment influenced the seasonal variation in the prevalence of STEC O157:H7.^37^

Ruminants are the reservoirs of *E. coli* O157:H7 and meat and milk contaminated with feces are the most common sources of human infection.^38^ High prevalence of *E. coli* O157:H7/NM has been reported in fecal samples from cattle, water buffalo, goat and sheep.^8^^,^^31^^,^^39^^,^^40^ Therefore, the contamination source of *E. coli* O157:H7/NM in retail raw meat in this study is likely to be insufficient hygiene during slaughter, transportation or handling and storage in butcheries.^41^

This study shows the importance of beef and water buffalo meat as potential sources of human *E. coli *O157:H7/NM infection. As the potential of contamination with *E. coli* O157:H7/NM can be considerable in slaughterhouses, the maintenance of slaughter hygiene and regular microbiological monitoring of carcasses are essential tools in minimizing the risk of direct and cross-contamination. Such risks especially exist when other species with lower prevalence of contamination are slaughtered at the same slaughtering line or stored at the same premises as those with higher predisposition to contamination. 
